# Supplementary material for: Hypoxia-induced miR-92a regulates p53 signaling pathway and apoptosis by targeting calcium-sensing receptor in genetically improved farmed tilapia (Oreochromis niloticus)
Source: PLoS One. 2020 Nov 12;15(11):e0238897. doi: 10.1371/journal.pone.0238897 (PMC7660578; doi:10.1371/journal.pone.0238897)
Supplement: S1 Fig — CaSR protein (66kD) was detected in GIFT liver sample. The procedures for SDS-PAGE preparation, protein sample electrophoresis, membrane transfer, blocking, and antibody incubation were as described by Qiang et al. [20]. Color was developed using Immobilon Western HRP substrate (Millipore, Billerica, MA, USA). (PDF) [file pone.0238897.s001.pdf]

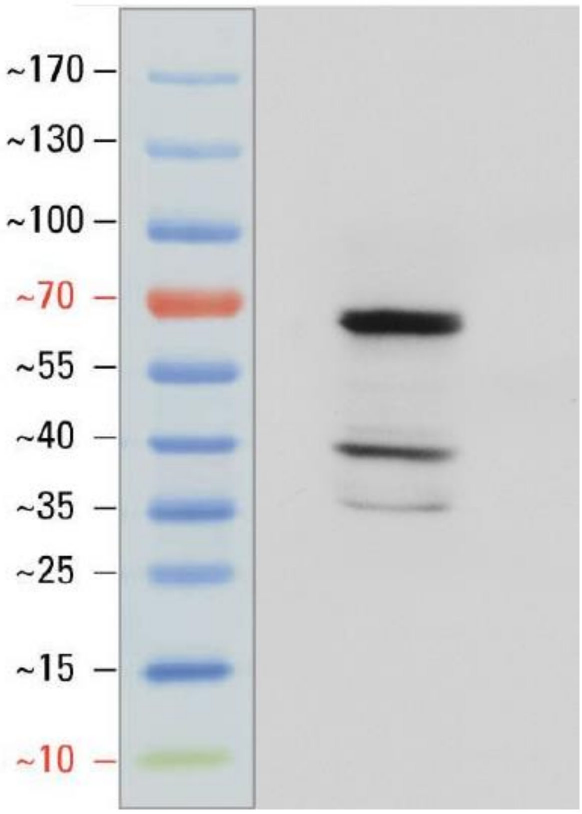

S1 Fig. Marker map of CaSR protein expression in GIFT.

CaSR protein (66kD) was detected in GIFT liver sample. The procedures for SDS-PAGE preparation, protein sample electrophoresis, membrane transfer, blocking, and antibody incubation were as described by Qiang et al. [20]. Color was developed using Immobilon Western HRP substrate (Millipore, Billerica, MA, USA).
