# Supplementary material for: Hypoxia-induced miR-92a regulates p53 signaling pathway and apoptosis by targeting calcium-sensing receptor in genetically improved farmed tilapia (Oreochromis niloticus)
Source: PLoS One. 2020 Nov 12;15(11):e0238897. doi: 10.1371/journal.pone.0238897 (PMC7660578; doi:10.1371/journal.pone.0238897)
Supplement: S2 Fig — CaSR protein (66kD) in miR-92a agomir group (A-D) was gradually weaken after 24 hours but was absent by 48 hours. CaSR protein (66kD) in PBS group (E-H) was used as a control and was present in liver samples. The procedures for SDS-PAGE preparation, protein sample electrophoresis, membrane transfer, blocking, and antibody incubation were as described by Qiang et al. [20]. Color was developed using Immobilon Western HRP substrate (Millipore, Billerica, MA, USA). (PDF) [file pone.0238897.s002.pdf]

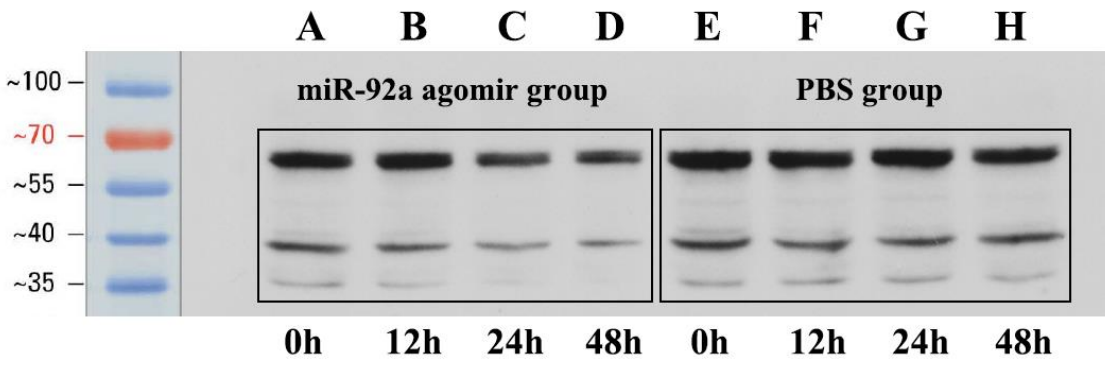

S2 Fig. Western blot of CaSR expression in GIFT both miR-92a agomir group and PBS group.

CaSR protein (66kD) in miR-92a agomir group (A-D) was gradually weaken after 24 hours but was absent by 48 hours. CaSR protein (66kD) in PBS group (E-H) was used as a control and was present in GIFT liver samples. The procedures for SDS-PAGE preparation, protein sample electrophoresis, membrane transfer, blocking, and antibody incubation were as described by Qiang et al. [20]. Color was developed using Immobilon Western HRP substrate (Millipore, Billerica, MA, USA).
